# Homœopathic Medical College of Missouri

**Published:** 1899-06

**Authors:** 


### HOMŒOPATHIC MEDICAL COLLEGE OF MISSOURI.

The forty-second annual commencement of the Homœopathic Medical College of Missouri took place on the evening of April 5th, at Y. M. C. A. Hall, Franklin and Grand Avenues, St. Louis. A large crowd was present. All the graduates wore the regulation cap and gown, the members of the faculty appearing in full dress. The exercises, which were very interesting, were opened, after the invocation, by a solo, "The Vagabond," by Dr. Malcolm Robb, which was followed by the report of the Dean, Dr. William G. Richardson. Miss Adah Block sang "A Song of Thanksgiving," after which Dr. James A. Campbell, President of the Board of Trustees, conferred the degree of Doctor of Medicine on the following graduates: C. A. Baldwin, Amboy, Ills.; Kate W. Beall, St. Louis; William Henry Bickley, Waterloo, Iowa; Edward Albert Bruno, Elkhart, Wis.; Mrs. Sara Isabel Barnard, St. Louis; Frank Willard Gillham, Brighton, Ills.; Joseph Adams Hirsch, Plymouth, Wis.; Augustus Stone Hunt, Jerseyville, Ills.; Fred L. Mitchell, Sibley, Iowa; Louis James Meurer, Chester, Ills.; Bertha Stark Pease, Hamilton, Ills.; Leslie C. Sammons, Dowagiac, Mich.; Charles Augustus Schreiner, Sigourney, Iowa.

After the conferring of degrees, Miss Helen Thorell rendered a violin solo, "Romance and Rondo," by Wienawski.

The "class honors" were then awarded by Frederick H. Bacon, the faculty prize being awarded to Joseph A. Hirsch, and honorable mention being given to Edward A. Bruno and Charles A. Schreiner. Miss Block and Mrs. Carl J. Luyties sang a duet, "Love," after which the address on behalf of the faculty was delivered by Rev. Cornelius H. Patton, D. D., the programme closing with a duo for harp and 'cello by Miss Adele Ghio and Mr. A. D. Woodward.

The commencement exercises were then closed by a banquet the following evening at the West End Hotel, given by the Alumni Association of the Homœopathic Medical College, and for which one hundred and thirty-five covers were laid.

